# Maternal Separation Modifies the Activity of Social Processing Brain Nuclei Upon Social Novelty Exposure

**DOI:** 10.3389/fnbeh.2021.651263

**Published:** 2021-11-04

**Authors:** Sara Mejía-Chávez, Arturo Venebra-Muñoz, Fabio García-García, Aleph Alejandro Corona-Morales, Arturo Enrique Orozco-Vargas

**Affiliations:** ^1^Laboratorio de Neurobiología de la Adicción y Plasticidad Cerebral, Facultad de Ciencias, Universidad Autónoma del Estado de Mexico, Toluca, Mexico; ^2^Laboratorio de Biología de Sueño, Instituto de Ciencias de la Salud, Universidad Veracruzana, Xalapa, Mexico; ^3^Laboratorio de Investigación Genómica, Universidad Veracruzana, Xalapa, Mexico; ^4^Centro Universitario Atlacomulco, Universidad Autónoma del Estado de Mexico, Atlacomulco, Mexico

**Keywords:** social novelty, nucleus accumbens, medial prefronatal cortex, reward, medial amygala, maternal separation

## Abstract

Maternal separation has been shown to disrupt proper brain development and maturation, having profound consequences on the neuroendocrine systems in charge of the stress response, and has been shown to induce behavioral and cognitive abnormalities. At the behavioral level, maternal separation has been shown to increase offensive play-fighting in juvenile individuals and reduce social interest in adulthood. Since most of the studies that have evaluated the consequences of maternal separation on social behavior have focused on behavioral analysis, there is a need for a further understanding of the neuronal mechanisms underlying the changes in social behavior induced by maternal separation. Therefore, the aim of the present research was to assess the long-term effects of maternal separation on social interaction behavior and to assess the activity of several brain regions involved in the processing of social cues and reward upon social novelty exposure, using c-Fos immunohistochemistry as a marker of neuronal activity. Male Wistar rats were subjected to 4 h maternal separation during the neonatal period, 9:00 h–13:00 h from postnatal day 1 to 21, and exposed to social novelty during adulthood. After social novelty exposure, brains were fixed and coronal sections of the medial amygdala, lateral septum (LS), paraventricular nucleus of the hypothalamus, nucleus accumbens, and medial prefrontal cortex were obtained for c-Fos immunohistochemistry. Maternally separated rats spent less time investigating the novel peer, suggesting that maternal separation reduces social approach motivation. Furthermore, maternal separation reduced the number of c-Fos positive cells of the medial amygdala, paraventricular nucleus of the hypothalamus, LS, nucleus accumbens, and medial prefrontal cortex upon social novelty exposure. These findings suggest that maternal separation can reduce the plastic capacity of several brain nuclei, which constitute a physiological basis for the emergence of behavioral disorders presented later in life reported to be linked to early life adversity.

## Introduction

Numerous studies have shown that early life adversity predisposes individuals to present psychopathologies with altered emotional, reward processing, and cognition deficits later in life (Costello et al., 2003; Green et al., 2010; Girardi et al., 2014; Birn et al., 2017; Novick et al., 2018), which are hypothesized to play a major role in the development of mental disorders (Dennison et al., 2017). In rodents, maternal separation has been widely used as early life adversity model, that consists of depriving pups of their mothers typically 3 h per day during the neonatal period (McEwan, 2000; Oitzl et al., 2000; Vetulani, 2013; Murthy and Gould, 2018). Studies have shown that maternal separation disrupts proper brain development and maturation, having profound consequences on the neuroendocrine systems in charge of the stress response (Levine, 2001; Vetulani, 2013), and has been shown to induce cognitive and behavioral abnormalities such as depressive (Ladd et al., 2000; Aisa et al., 2007) and anxiety-like behaviors (Wigger and Neumann, 1999; Huot et al., 2001; Salm et al., 2004; Aisa et al., 2007), anhedonia (Huot et al., 2001), alterations in reward processing and cognitive functions (McEwan, 2000). Furthermore, maternal separation has also been reported to induce structural expansion and increase the excitability of the amygdala (Koe et al., 2016), reduce the expression of brain-derived neurotrophic factor (BDNF) in the medial prefrontal cortex (mPFC) and nucleus accumbens (NAc) in adult rats (Wang et al., 2015), indicating that maternal separation can influence neuroplasticity in different ages. Age-specific changes in the brain induced by maternal separation have also been reported in other animals. For example, parental deprivation of the biparental rodent Octodon degus suppresses synapse formation and dendritic growth within the orbitofrontal cortex in adults, but not in juvenile individuals (Helmeke et al., 2009).

There are specific brain regions in charge of the processing of social information, which is referred to, by some authors, as the *social brain* (Insel and Fernald, 2004), and central to the understanding of these brain regions is an insight into the distribution of neuronal activity that drives social behavior (Kim et al., 2015). One way of evaluating neuronal activity is by the detection of immediate early genes (IEGs) or IEG products, such as c-Fos (Kaczmarek et al., 1988; Sheng and Greenberg, 1990; Guzowski et al., 2005; Jaworski et al., 2018), whose expression has been linked to neuronal activity (Morgan et al., 1987; Kaczmarek et al., 1988; Jaworski et al., 2018). Therefore, its mapping can provide a view of recent whole-brain activity (Clayton, 2000; Guzowski et al., 2005).

In a recent study, we compared the number of c-Fos positive cells in juvenile rats after the exposure to social familiarity, non-social physical novelty, and social novelty in different brain nuclei, and showed that social novelty exposure induces the expression of a higher number of c-Fos positive cells in the paraventricular nucleus of the hypothalamus (PVN) and nuclei that comprise the reward system such as the NAc and ventral pallidum (VP), suggesting that social novelty during youth is a more highly rewarding stimulus compared with social familiarity and non-social physical novelty (Gómez-Gómez et al., 2019).

Concerning maternal separation and social behavior, maternal separation has been shown to increase offensive play-fighting in juvenile individuals; maternally separated individuals exhibited an increase in the number of nape attacks, a higher frequency of offensive pulling and biting towards their opponents, and exhibit lower frequency of submissive play behaviors (Veenema and Neumann, 2009). Furthermore, some studies have reported that maternal separation alters other social behaviors, such as sexual behavior and aggression in adult male rats (Rhees et al., 2001; Greisen et al., 2005; Veenema et al., 2006), and reduces social interaction behavior in juvenile and adult individuals (Lukas et al., 2011; Girardi et al., 2014; Houwing et al., 2019; Lee and Han, 2019; Wei et al., 2020). These findings show that maternal separation modifies the way individuals cope with social encounters throughout life. However, there is a need for a further understanding of the neuronal and molecular mechanisms underlying the changes in the social behavior of adult individuals induced by maternal separation (Kentrop et al., 2018; Kambali et al., 2019; Wei et al., 2020). The aim of the present research was to assess the long-term effects of maternal separation on social interaction behavior and to assess the activity of several brain regions involved in the processing of social cues and reward upon social novelty exposure, using c-Fos as a marker of neuronal activity.

## Materials and Methods

### Animals

Forty male Wistar rats were used. Animal handling and internal bioterium conditions were applied in accordance with the official norm NOM-062-ZOO-1999 (NORMA Oficial Mexicana: NOM-062-ZOO-1999, 1999) standards and the Guide for the Care and Use of Laboratory Animals (National Research Council, 2011). Every effort was made to minimize animals’ discomfort. Subjects were obtained *via* controlled crossbreeding to ascertain their litter of provenance. Male breeder rats were removed before pups were born when the dam first appeared pregnant. Rats were divided into two groups: control subjects (CS, *n* = 20) and maternal separation group (MS, *n* = 20). These two groups were further divided into two sub-groups as follows: (1) CS + No Test (CS-NT, *n* = 10); (2) CS + Social Novelty (CS-SN, *n* = 10); (3) MS + No Test (MS-NT, *n* = 10); MS + Social Novelty (MS-SN, *n* = 10). The procedure for each experimental group is described below.

### Maternal Separation

Maternal separation was conducted as described by Wang et al. (2015). Pups were separated from their dams for 4 h daily during the light phase of the light cycle (9:00 h–13:00 h) from postnatal day 1 to 21. During separation, pups were placed in cages containing clean bedding in groups of four so that they could maintain their body temperature. Following the 4 h separation, pups were returned to their respective home cage and reunited with their dam until the maternal separation of the following day.

### Social Novelty Test

Social novelty tests were performed in the test area described by Gómez-Gómez et al. (2019). The test area was a standard bioterium polycarbonate cage (45 cm long, 25 cm wide, and 20 cm high) with a top metal mesh with 0.5 cm^2^ cells, and a metal mesh with 1.3 cm^2^ cells in the middle of the cage. The purpose of the mesh placed in the middle of the cage was to avoid body-to-body contact, aggressive behaviors, and play, but allowed olfaction between individuals, and slight body-to-body contact. Individuals subjected to the social novelty test were habituated to the test area on postnatal day 52; rats were individually placed in the test area for 30 min and then returned to their respective home cages until the following day. All social novelty tests were carried out on postnatal day 53. Rats were exposed to a novel peer of the same age reared in a different litter; subjects were individually placed on each side of the mesh and were left to interact for 15 min. Tests were conducted during the dark phase of the light cycle, under red lighting, between 10:00 h and 13:00 h. All tests were recorded for further behavioral analysis.

### Procedure for Control Subjects—No Test Group

Pups were raised in standard bioterium cages with their dam until weaning on postnatal day 21. On postnatal day 21 rats were weaned and housed in individual cages, with food and water *ad libitum* and left undisturbed until postnatal day 53. On postnatal day 53, transcardial perfusion was performed for the fixation of the brain.

### Procedure for Maternal Separation—No Test Group

Maternal separation was conducted for this group. On postnatal day 21, rats were weaned and housed in individual cages, with food and water *ad libitum* and left undisturbed until postnatal day 53. On postnatal day 53, transcardial perfusion was performed for the fixation of the brain.

### Procedure for Control Subjects—Social Novelty Group

Pups were raised in standard bioterium cages with their dam until weaning on postnatal day 21. On postnatal day 21, rats were housed in individual cages, with food and water *ad libitum* and left undisturbed until postnatal day 52. On postnatal day 52, rats were individually placed in the social novelty test area for 30 min to habituate the subjects with the test cage. Following this, individuals were returned to their respective cages until the following day. Social novelty tests were carried out on postnatal day 53. Following this, animals were returned to their respective cages for 1 h and transcardial perfusion was performed for the fixation of the brain.

### Procedure for Maternal Separation—Social Novelty Group

**Figure 1:**
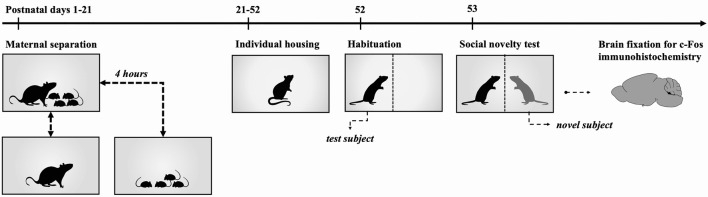
Timeline for Maternal Separation-Social Novelty group. Experimental rats were subjected to 4 h maternal separation from postnatal days 1 to 21. On postnatal day 21 subjects were weaned and housed in individual cages with food and water *ad libitum* until postnatal day 52. On postnatal day 52, rats were individually placed in the social novelty test area for 30 min to habituate the subjects to the test cage. Following this, individuals were returned to their cages until the following day. Social novelty tests were carried out on postnatal day 53. After social novelty exposure, animals were returned to their respective cages for 1 h and transcardial perfusion was performed for the fixation of the brain.

The procedure for this group was the same as for the CS-SN group, except that maternal separation was conducted (Figure 1).

### Behavioral Analysis

Social interaction and self-grooming measures were taken during the first 5 min of the social interaction test. Social interaction was considered as the total time the test rat spent sniffing or whisking the novel rat, and when there was slight body-to-body contact between individuals. The test rat was considered to be sniffing the novel peer when the individual made rapid twitching movements of the nose, either with the nose in contact with the novel subject or held in an elevated position in the air facing the novel peer when the distance between the nose tip and metal mesh was 1 cm or less. Whisking was considered as the contact between the whiskers of the test rat and the novel subject, or when the test rat brushed its whiskers along the body surface of the novel peer. The social latency approach refers to the latency time to the occurrence of social interaction. The social approach was considered as the total number of times the test subject approached the novel peer. Behavioral data was assessed for normal distribution using the Kolmogorov–Smirnov normality test (*α* = 0.05). Student’s *t-*test was used for the analysis of the behavioral data of groups CS-SN and MS-SN. Significance for all analyses was defined as *p* < 0.05.

### Immunohistochemistry

**Figure 2:**
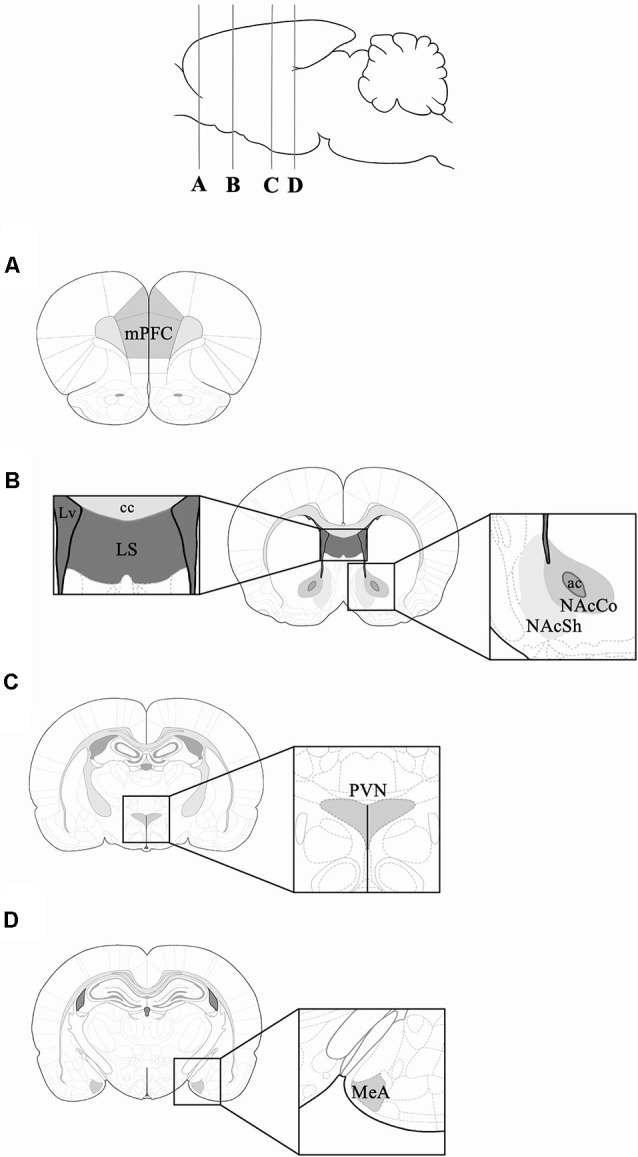
Schematic representation of the coronal rat brain sections obtained for c-Fos immunohistochemistry. Coronal sections corresponding to: **(A)** mPFC (Bregma 3.72 mm); **(B)** lateral septum (LS), NAcSh and NAcCo (Bregma 1.20 mm); **(C)** PVN (Bregma −1.92 mm); and **(D)** MeA (Bregma −3.00 mm). mPFC, medial Prefrontal Cortex; MeA, Medial Amygdala; NAcSh, Nucleus Accumbens Shell; NAcCo, Nucleus Accumbens Core; PVN, Paraventricular Nucleus; Lv, lateral ventricle; cc, corpus callosum; ac, anterior commissure. Rat brain and coronal rat brain section illustrations were adapted from Paxinos and Watson (2007).

Rats were deeply anesthetized with sodium pentobarbital intraperitoneally and transcardially perfused with saline solution (0.9%), followed by paraformaldehyde (4%) in 0.1 M phosphate buffer (PB) pH 7.4. Brains were removed, postfixed overnight, and then equilibrated to a gradient of sucrose solutions (10, 20, and 30%, 24 h each). Coronal sections of the medial amygdala (MeA; Bregma: −2.70 to −3.20), paraventricular nucleus of the hypothalamus (PVN; Bregma: −1.30 to −1.60 mm), lateral septum (LS), nucleus accumbens shell (NAcSh), nucleus accumbens core (NAcCo; Bregma: 1.60 to 1.20 mm) and medial prefrontal cortex (mPFC; Bregma: 4.20 to 3.20 mm) were obtained (Paxinos and Watson, 2007; Figure 2). These brain regions were selected because they are implicated in social behavior and reward (O’Connell and Hoffman, 2011; Love, 2013). Tissue sections were incubated in 0.3% hydrogen peroxide for 10 min, and then incubated in PB, 0.3% Triton X-100, and 3% normal goat serum for an hour. Then, tissue sections were incubated in the same solution with the addition of the c-Fos antibody (sc-52; Santa Cruz Biotechnology, Santa Cruz, California, USA) at a 1:250 dilution for 2 days at 4°C. Tissue sections were incubated in a secondary antibody (biotinylated goat anti-rabbit, sc-2040; Santa Cruz Biotechnology), a dilution of 1:250 in PB with 0.3% Triton X-100, and 3% normal goat serum for an hour at room temperature. The reaction was visualized with a solution of PB with 0.06% diaminobenzidine, 1% nickel sulfate, and 1% cobalt chloride. Some sections from each experimental group were processed as described above, but without the primary antibody, as negative control groups for the immunohistochemistry.

### Quantification of c-Fos Positive Cells

c-Fos-positive cells were identified as a black-purple precipitate from the DAB-nickel/cobalt reaction in the cell nucleus. All slides were coded and c-Fos positive cells were counted in both hemispheres using an optic microscope with a 40x objective (64,310 μm^2^ per hemisphere). Four sections per structure were analyzed. All data were assessed for normal distribution using the Kolmogorov–Smirnov normality test (*α* = 0.05). One-way ANOVA’s and Tuckey *post hoc* tests per area were applied to determine significant differences in the number of c-Fos positive cells between the experimental groups. Significance for all analyses was defined as *p* < 0.05. All data are presented as mean ± SEM.

## Results

### Behavior

**Figure 3:**
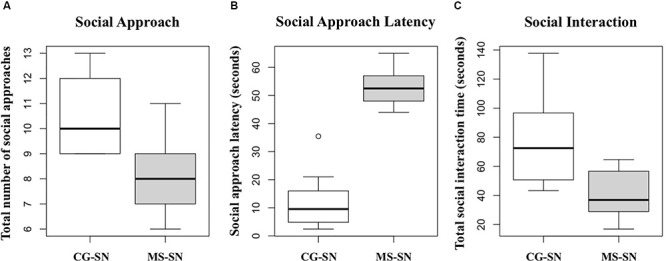
Boxplots for the data of: **(A)** Total number of social approaches. **(B)** Social approach latency. **(C)** Total social interaction time. CG-SN (*n* = 10), Control Subjects-Social Novelty group; MS-SN (*n* = 10), Maternal Separation-Social Novelty group. Student’s *t-*test was used to compare behavioral data between groups CS-SN and MS-SN. Significance for all analyses was defined as *p* < 0.05.

Student’s *t-*test showed that, compared to non-maternally separated rats, the number of social approaches was significantly lower in maternally separated rats (*t* = 3.85, *P* < 0.05; Figure 3A), latency to approach the novel peer was higher in maternally separated rats (*t* = 10.81, *P* < 0.05; Figure 3B) and they spent significantly less time interacting with the novel conspecific (*t* = 3.44, *P* < 0.05; Figure 3C).

### c-Fos Positive Cells in the Medial Amygdala

**Figure 4:**
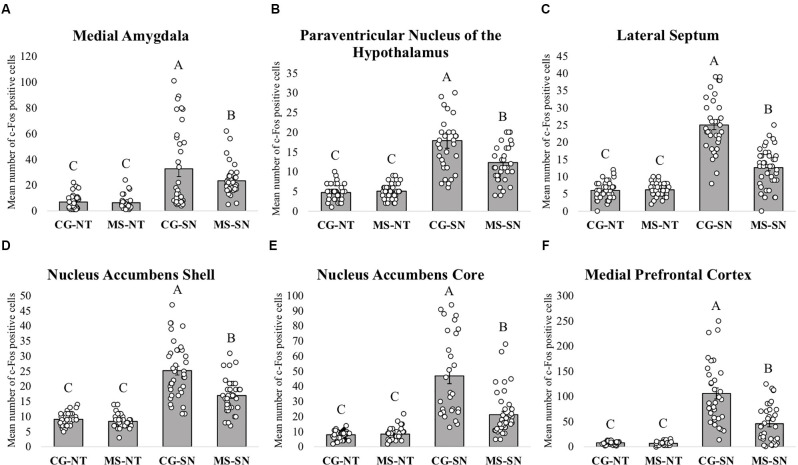
Immunohistochemistry analysis. The graphs show the mean number of c-Fos positive cells in the different groups for each brain area ± SEM. The letters A, B, and C above the bars indicate statistically significant differences between groups in accordance with the *post hoc* Tuckey tests. c-Fos positive cells were counted using an optic microscope with a 40x objective (64,310 μm^2^ per hemisphere). **(A)** Mean number of c-Fos positive cells in the Medial Amygdala. **(B)** Mean number of c-Fos positive cells in the Paraventricular Nucleus of the Hypothalamus. **(C)** Mean number of c-Fos positive cells in the LS. **(D)** Mean number of c-Fos positive cells in the Nucleus Accumbens Shell. **(E)** Mean number of c-Fos positive in the Nucleus Accumbens Core. **(F)** Mean number of c-Fos positive cells in the medial Prefrontal Cortex. CS-NT, Control Subjects-No Test group; MS-NT, Maternal Separation-No Test group; CS-SN, Control Subjects-Social Novelty Group; MS-SN, Maternal Separation-Social Novelty group. All data are presented as mean ± SEM.

**Figure 5:**
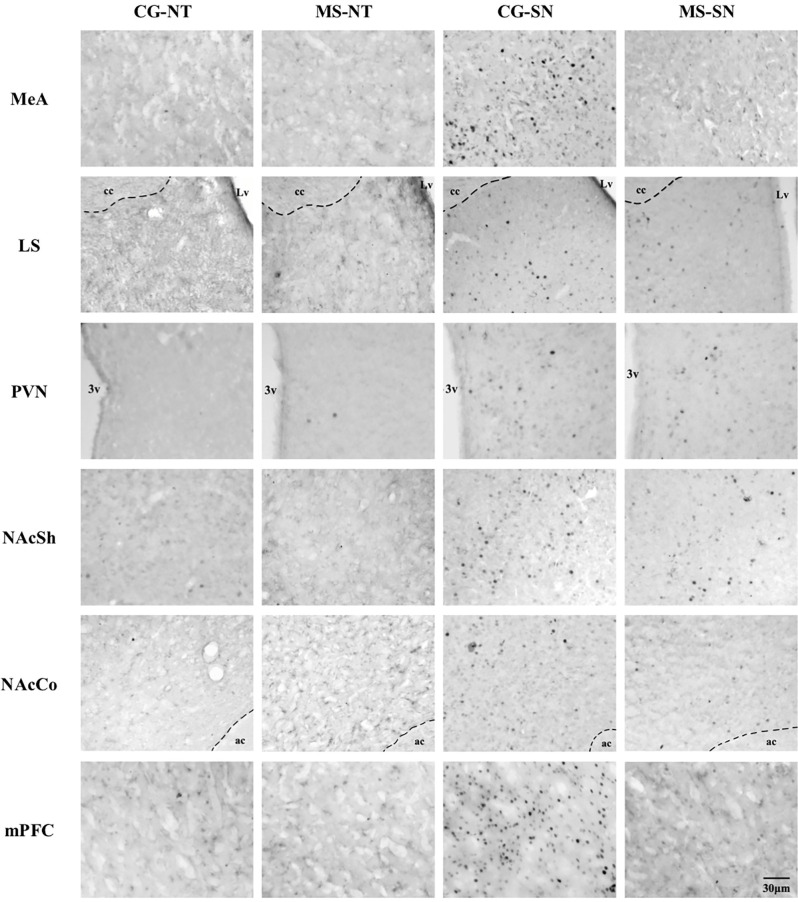
Microphotographs of the analyzed brain sections for c-Fos positive cells. Columns represent the experimental groups (CS-NT, MS-NT, CS-SN, and MS-SN); rows represent the brain areas (MeA, LS, PVN, NAcSh, NAcCo, and mPFC). The scale bar represents 30 μm for all photographs. CS-NT, Control Subjects-No Test group; MS-NT, Maternal Separation-No Test group; CS-SN, Control Subjects-Social Novelty Group; MS-SN, Maternal Separation-Social Novelty group; MeA, Medial Amygdala; LS, Lateral Septum; PVN, paraventricular nucleus; NAc, Nucleus Accumbens, NAcSh, Nucleus Accumbens Shell; NAcCo, Nucleus Accumbens Core; mPFC, medial Prefrontal Cortex; 3v, third ventricle; Lv, lateral ventricle; cc, corpus callosum; ac, anterior commissure.

Statistically significant differences in the number of c-Fos positive cells between CS-SN and MS-SN groups, compared with CS-NT and MS-NT groups were found (*F*_(3,150)_ = 22.02, *P* < 0.05; Figures 4A, 5). The number of cells was significantly higher in groups exposed to social novelty CS-SN and MS-SN compared with control groups CS-NT and MS-NT, but the number of c-Fos positive neurons was significantly lower in maternally separated rats exposed to social novelty MS-SN compared with non-maternally separated rats exposed to social novelty CS-SN.

### c-Fos Positive Cells in the Lateral Septum

The number of c-Fos positive cells was significantly higher in groups exposed to social novelty CS-SN and MS-SN compared with the control groups CS-NT and MS-NT (*F*_(3,149)_ = 110.51, *P* < 0.05; Figures 4C, 5), but the number of c-Fos positive cells was significantly lower in maternally separated rats exposed to social novelty MS-SN compared with non-maternally separated rats exposed to social novelty CS-SN.

### c-Fos Positive Cells in the Paraventricular Nucleus of the Hypothalamus

The mean number of c-Fos positive cells of the experimental groups exposed to social novelty (CS-NS and MS-NS) was significantly higher compared to the control groups CS-NT and MS-NT (*F*_(3,138)_ = 35.31, *P* < 0.05), but maternally separated individuals showed a significantly lower number of c-Fos positive cells compared to non-maternally separated subjects exposed to social novelty (Figures 4B, 5).

### c-Fos Positive Cells in the Nucleus Accumbens

The number of c-Fos positive cells were counted in the two areas that comprise the NAc: NAcSh and NAcCo. For both areas, the number of c-Fos positive cells was significantly higher in groups exposed to social novelty CS-SN and MS-SN compared with control groups CS-NT and MS-NT, but the number of c-Fos positive neurons was significantly lower in maternally separated rats exposed to social novelty MS-SN compared with non-maternally separated rats exposed to social novelty CS-SN (NAcSh: *F*_(3,148)_ = 74.26, *P* < 0.05, Figure 4D; NAcCo: *F*_(3,149)_ = 51.73, *P* < 0.05, Figures 4E, 5).

### c-Fos Positive Cells in the Medial Prefrontal Cortex

The number of c-Fos positive cells was significantly higher in groups exposed to social novelty CS-SN and MS-SN compared with the control groups CS-NT and MS-NT (*F*_(3,123)_ = 47.16, *P* < 0.05; Figure 4F).

## Discussion

In the present research, the maternal separation procedure was based on the study by Wang et al. (2015), in which they found differences in the expression of BDNF in brain regions that were also analyzed here, such as the NAc and mPFC of adult but not juvenile rats maternally separated rats. Additionally, Lukas et al. (2011) showed that adult maternally separated rats failed to discriminate between a previously encountered and novel rat and that the social recognition impairment of maternally separated rats was accompanied by a lack of a rise in vasopressin release within the LS during social memory acquisition. These studies made us suspect that the changes in social behavior induced by maternal separation in adult individuals (Veenema and Neumann, 2009; Lukas et al., 2011; Girardi et al., 2014; Lee and Han, 2019; Houwing et al., 2019) could be accompanied by changes in neuronal activation of brain regions involved in the social processing of social cues. Therefore, the aim of the present research was to assess the long-term effects of maternal separation on social interaction behavior of adult rats and to assess the activity of several brain regions involved in the processing of social cues and reward upon social novelty exposure, using c-Fos as a marker of neuronal activity.

### Behavior

Exposure to chronic stress, such as maternal separation, has been reported to suppress motivation to participate in pleasurable activities (Birn et al., 2017; Dennison et al., 2017). Social interactions are labeled as highly rewarding stimuli in both humans and animals since they can instill a sense of well-being and pleasure and motivate approach behaviors towards specific social stimuli (Trezza et al., 2011). Furthermore, social interactions are more highly rewarding during youth (Gómez-Gómez et al., 2019) since individuals seek out social interactions more frequently during this life stage than their adult counterparts (Smith et al., 2015). There are controversial reports on the effects of maternal care separation on social behavior (Girardi et al., 2014; Lee and Han, 2019; Houwing et al., 2019). However, the different results could be due to differences in maternal separation procedures and the age of the subjects at which the different social behavioral tests were performed in each study. For example, 22 h of maternal separation on postnatal day 10 reduces social investigation in young individuals (Girardi et al., 2014), while 24 h of maternal separation on postnatal day 3 and 6 h of maternal separation during postnatal days 4–14 does not modify social interaction behavior in adulthood (Kentrop et al., 2018; Kambali et al., 2019). The results of the present research are in accordance with the studies that show that maternal separation reduces social behavior (Girardi et al., 2014; Lee and Han, 2019; Houwing et al., 2019), and the fact that maternally separated rats spent less time socializing with the novel peer suggests that maternal care separation reduces the expression of behaviors related to social reward, possibly due to morphological and physiological changes in brain regions involved in reward processing (Dennison et al., 2017), as we discuss below. Furthermore, the view that maternally separated individuals exhibited an increased latency to approach the novel peer and spent less time interacting with the novel conspecific suggests higher anxiety levels in maternally separated rats during social novelty exposure (File, 1985; Campos et al., 2013; Lezak et al., 2017).

### c-Fos Positive Cells in Social Processing Brain Nuclei

Studies have shown that the MeA plays a crucial role in the processing of novel social information since its activity increases when individuals interact with a novel peer (Ferguson et al., 2001; Kim et al., 2015), mainly by oxytocin receptor activation (Ferguson et al., 2001). Early-life stress has been shown to have persistent effects on amygdala circuitry and function (Cohen et al., 2013; Koe et al., 2016). Therefore, the reduced neuronal activity in the MeA of maternally separated individuals could be due to a blunted oxytocin receptor activation during social novelty exposure, However, it is necessary to confirm that the changes in c-Fos expression are occurring specifically in oxytocin receptor-expressing neurons. A projection site of the MeA relevant in the processing of social information and reward is the LS (Caffé et al., 1987; de Vries, 2008). A study that evaluated the endogenous release of vasopressin within the LS showed that maternal separation impairs social recognition in adult individuals since it reduces the release of vasopressin within the LS during social memory acquisition (Lukas et al., 2011). Given the results of this study, the reduced number of c-Fos positive cells in the LS of maternally separated individuals following social novelty exposure could be due to the blunted release of vasopressin within this brain region (Lukas et al., 2011), therefore indicate that septal vasopressin release in adult individuals during novel social encounters is lessened upon early life stress exposure.

In a previous study, we showed that the activity of the PVN increases when juvenile rats are exposed to social novelty (Gómez-Gómez et al., 2019). Similarly, the results of the present study show that the activity of the PVN increases upon social novelty exposure in adult individuals but is affected by maternal separation (Figure 5). It has been shown that PVN oxytocinergic activity increases during social interactions (Hung et al., 2017), and oxytocinergic neurons in the PVN project to the mPFC (Knobloch et al., 2012) and NAc (Ross et al., 2009). Since the number of c-Fos positive cells was reduced in maternally separated individuals, we hypothesize that maternal separation reduces the activity of oxytocinergic neurons in the PVN upon social novelty exposure, although a c-Fos/oxytocin double labeling would be necessary to confirm this.

The findings of the present study show that upon social novelty exposure, maternal separation disrupts the activity of the NAc of adult rats since the number of c-Fos positive cells was lower in maternally separated rats. We suggest that the reduced activity of the nucleus accumbens of maternally separated rats could be related to the blunted activity of other brain regions that have direct projections to this brain region such as the MeA, LS (O’Connell and Hoffman, 2011), the PVN (Dölen et al., 2013), and possibly dopaminergic neurons of the ventral tegmental area (VTA) known to play an important role in social reward (Gunaydin et al., 2014; Hung et al., 2017; Peris et al., 2017). Our findings show that maternally separated rats spent less time investigating a novel peer and showed a reduced activity of the NAc. This shows that maternal separation reduces social interaction behavior due to changes in the activity of the NAc, and possibly other brain regions involved in reward processing such as the VP and VTA (Smith and Berridge, 2007; Peris et al., 2017).

In a previous study, we showed that activity of the NAc and VP increases upon social novelty exposure and that social novelty is a more rewarding stimulus (Gómez-Gómez et al., 2019). It has been shown that the exposure to adverse events during early life stages induces changes in the microstructural properties of brain regions involved in reward processing (Dennison et al., 2017), including the NAc, and mPFC (Wang et al., 2015), and that such changes have been related to alterations in reward processing (Birn et al., 2017; Dennison et al., 2017). Since the mPFC, along with the NAc and VTA, have been proposed as the key neural substrate for the regulation of social behavior and social motivation (Gunaydin et al., 2014; Kas et al., 2014; Kim et al., 2015), the reduction of the activity of the NAc and mPFC shows that maternal separation reduces the hedonic value of social interactions in adulthood. Although to delve into this topic, the activity of the VTA and VP should also be considered. Finally, since c-Fos has been suggested as an indicator of neuronal plasticity (Jaworski et al., 2018), the results of the present research showed that maternal separation can affect the plastic capacity of several brain nuclei, which constitute a physiological basis for the emergence of behavioral disorders presented later in life reported to be linked to early life adversity (Costello et al., 2003; Green et al., 2010; Girardi et al., 2014; Birn et al., 2017).

## Data Availability Statement

The original contributions presented in the study are included in the article/Supplementary Material, further inquiries can be directed to the corresponding author.

## Ethics Statement

The animal study was reviewed and approved by Norma Official Mexicana 062.

## Author Contributions

SM-C designed and performed the research under the supervision of AV-M. SM-C and AV-M analyzed the data and prepared the manuscript. FG-G, AC-M, and AO-V reviewed the manuscript. All authors contributed to the article and approved the submitted version.

## Conflict of Interest

The authors declare that the research was conducted in the absence of any commercial or financial relationships that could be construed as a potential conflict of interest.

## Publisher’s Note

All claims expressed in this article are solely those of the authors and do not necessarily represent those of their affiliated organizations, or those of the publisher, the editors and the reviewers. Any product that may be evaluated in this article, or claim that may be made by its manufacturer, is not guaranteed or endorsed by the publisher.

## Abbreviations

3v: third ventricle
ac: anterior commissure
BDNF: Brain Derived Neurotrophic Factor
cc: corpus callosum
IEGs: Immediate-Early Genes
LS: Lateral Septum
Lv: lateral ventricle
MeA: Medial Amygdala
mPFC: medial Prefrontal Cortex
NAc: Nucleus Accumbens
NAcCo: Nucleus Accumbens Core
NAcSh: Nucleus Accumbens Shell
PVN: Paraventricular Nucleus of the Hypothalamus
SEM: Standard Error of the Mean
VP: Ventral Pallidum
VTA: Ventral Tegmental Area.
