# Supplementary material for: Maternal Separation Modifies the Activity of Social Processing Brain Nuclei Upon Social Novelty Exposure
Source: Front Behav Neurosci. 2021 Nov 4;15:651263. doi: 10.3389/fnbeh.2021.651263 (PMC8599987; doi:10.3389/fnbeh.2021.651263)
Supplement: Supplementary file 1 [file Table_1.docx]

Supplementary Material

# Supplementary Datasets

The datasets generated and analyzed for the present research are presented in the following tables.

**Table 1.** Dataset of the number of c-Fos immunoreactive cells in the medial amygdala of the different experimental groups.

| Group | Number of c-Fos positive cells | Group | Number of c-Fos positive cells |
| --- | --- | --- | --- |
| CG-NT | 2 | CG-SN | 57 |
| CG-NT | 3 | CG-SN | 101 |
| CG-NT | 3 | CG-SN | 89 |
| CG-NT | 1 | CG-SN | 59 |
| CG-NT | 2 | CG-SN | 53 |
| CG-NT | 2 | CG-SN | 79 |
| CG-NT | 2 | CG-SN | 79 |
| CG-NT | 6 | CG-SN | 118 |
| CG-NT | 1 | CG-SN | 34 |
| CG-NT | 1 | CG-SN | 87 |
| CG-NT | 4 | CG-SN | 120 |
| CG-NT | 4 | CG-SN | 71 |
| CG-NT | 1 | CG-SN | 52 |
| CG-NT | 3 | CG-SN | 38 |
| CG-NT | 2 | CG-SN | 46 |
| CG-NT | 10 | CG-SN | 80 |
| CG-NT | 9 | CG-SN | 9 |
| CG-NT | 6 | CG-SN | 8 |
| CG-NT | 11 | CG-SN | 8 |
| CG-NT | 4 | CG-SN | 8 |
| CG-NT | 4 | CG-SN | 10 |
| CG-NT | 7 | CG-SN | 5 |
| CG-NT | 4 | CG-SN | 6 |
| CG-NT | 7 | CG-SN | 13 |
| CG-NT | 9 | CG-SN | 22 |
| CG-NT | 6 | CG-SN | 7 |
| CG-NT | 19 | CG-SN | 7 |
| CG-NT | 10 | CG-SN | 8 |
| CG-NT | 3 | CG-SN | 7 |
| CG-NT | 22 | CG-SN | 11 |
| CG-NT | 8 | CG-SN | 4 |
| CG-NT | 18 | CG-SN | 5 |
| CG-NT | 10 | CG-SN | 6 |
| CG-NT | 12 | CG-SN | 13 |
| CG-NT | 6 | CG-SN | 7 |
| CG-NT | 11 | CG-SN | 7 |
| CG-NT | 10 | MS-SN | 30 |
| CG-NT | 10 | MS-SN | 5 |
| CG-NT | 17 | MS-SN | 19 |
| CG-NT | 7 | MS-SN | 19 |
| MS-NT | 5 | MS-SN | 62 |
| MS-NT | 8 | MS-SN | 45 |
| MS-NT | 2 | MS-SN | 56 |
| MS-NT | 6 | MS-SN | 40 |
| MS-NT | 3 | MS-SN | 24 |
| MS-NT | 5 | MS-SN | 19 |
| MS-NT | 6 | MS-SN | 19 |
| MS-NT | 3 | MS-SN | 12 |
| MS-NT | 1 | MS-SN | 21 |
| MS-NT | 4 | MS-SN | 26 |
| MS-NT | 5 | MS-SN | 17 |
| MS-NT | 2 | MS-SN | 20 |
| MS-NT | 6 | MS-SN | 26 |
| MS-NT | 7 | MS-SN | 20 |
| MS-NT | 5 | MS-SN | 13 |
| MS-NT | 2 | MS-SN | 20 |
| MS-NT | 8 | MS-SN | 16 |
| MS-NT | 17 | MS-SN | 18 |
| MS-NT | 5 | MS-SN | 36 |
| MS-NT | 6 | MS-SN | 17 |
| MS-NT | 24 | MS-SN | 16 |
| MS-NT | 13 | MS-SN | 19 |
| MS-NT | 3 | MS-SN | 24 |
| MS-NT | 13 | MS-SN | 22 |
| MS-NT | 4 | MS-SN | 20 |
| MS-NT | 7 | MS-SN | 24 |
| MS-NT | 6 | MS-SN | 19 |
| MS-NT | 18 | MS-SN | 17 |
| MS-NT | 1 | MS-SN | 28 |
| MS-NT | 3 | MS-SN | 27 |
| MS-NT | 7 | MS-SN | 30 |
| MS-NT | 4 | MS-SN | 27 |
| MS-NT | 4 | MS-SN | 23 |
|  |  | MS-SN | 16 |
|  |  | MS-SN | 22 |
|  |  | MS-SN | 6 |
|  |  | MS-SN | 17 |

The table shows the total number of c-Fos immunoreactive cells counted in the medial amygdala in both hemispheres of each rat brain coronal section. CS-NT, Control Subjects-No Test group; MS-NT, Maternal Separation-No Test group; CS-SN, Control Subjects-Social Novelty Group; MS-SN, Maternal Separation-Social Novelty group.

**Table 2.** Dataset of the number of c-Fos immunoreactive cells in the nucleus accumbens shell of the different experimental groups.

| Group | Number of c-Fos positive cells | Group | Number of c-Fos positive cells |
| --- | --- | --- | --- |
| CG-NT | 10 | CG-SN | 13 |
| CG-NT | 11 | CG-SN | 20 |
| CG-NT | 9 | CG-SN | 31 |
| CG-NT | 8 | CG-SN | 19 |
| CG-NT | 8 | CG-SN | 23 |
| CG-NT | 8 | CG-SN | 39 |
| CG-NT | 9 | CG-SN | 40 |
| CG-NT | 10 | CG-SN | 14 |
| CG-NT | 10 | CG-SN | 22 |
| CG-NT | 8 | CG-SN | 25 |
| CG-NT | 6 | CG-SN | 19 |
| CG-NT | 12 | CG-SN | 16 |
| CG-NT | 9 | CG-SN | 27 |
| CG-NT | 11 | CG-SN | 11 |
| CG-NT | 6 | CG-SN | 23 |
| CG-NT | 7 | CG-SN | 11 |
| CG-NT | 8 | CG-SN | 32 |
| CG-NT | 9 | CG-SN | 28 |
| CG-NT | 11 | CG-SN | 25 |
| CG-NT | 7 | CG-SN | 32 |
| CG-NT | 7 | CG-SN | 41 |
| CG-NT | 9 | CG-SN | 47 |
| CG-NT | 9 | CG-SN | 21 |
| CG-NT | 7 | CG-SN | 35 |
| CG-NT | 11 | CG-SN | 30 |
| CG-NT | 14 | CG-SN | 21 |
| CG-NT | 9 | CG-SN | 24 |
| CG-NT | 5 | CG-SN | 26 |
| CG-NT | 13 | CG-SN | 13 |
| CG-NT | 11 | CG-SN | 30 |
| CG-NT | 7 | CG-SN | 21 |
| CG-NT | 9 | CG-SN | 17 |
| CG-NT | 13 | CG-SN | 32 |
| CG-NT | 8 | CG-SN | 41 |
| MS-NT | 9 | CG-SN | 20 |
| MS-NT | 7 | CG-SN | 18 |
| MS-NT | 8 | CG-SN | 33 |
| MS-NT | 3 | CG-SN | 17 |
| MS-NT | 7 | CG-SN | 26 |
| MS-NT | 8 | MS-SN | 22 |
| MS-NT | 7 | MS-SN | 14 |
| MS-NT | 8 | MS-SN | 21 |
| MS-NT | 8 | MS-SN | 27 |
| MS-NT | 8 | MS-SN | 19 |
| MS-NT | 9 | MS-SN | 12 |
| MS-NT | 7 | MS-SN | 7 |
| MS-NT | 7 | MS-SN | 8 |
| MS-NT | 8 | MS-SN | 17 |
| MS-NT | 6 | MS-SN | 10 |
| MS-NT | 7 | MS-SN | 8 |
| MS-NT | 11 | MS-SN | 10 |
| MS-NT | 9 | MS-SN | 13 |
| MS-NT | 14 | MS-SN | 13 |
| MS-NT | 7 | MS-SN | 15 |
| MS-NT | 14 | MS-SN | 16 |
| MS-NT | 6 | MS-SN | 27 |
| MS-NT | 9 | MS-SN | 17 |
| MS-NT | 10 | MS-SN | 19 |
| MS-NT | 9 | MS-SN | 31 |
| MS-NT | 7 | MS-SN | 19 |
| MS-NT | 7 | MS-SN | 12 |
| MS-NT | 9 | MS-SN | 16 |
| MS-NT | 10 | MS-SN | 19 |
| MS-NT | 8 | MS-SN | 21 |
| MS-NT | 12 | MS-SN | 13 |
| MS-NT | 9 | MS-SN | 17 |
| MS-NT | 8 | MS-SN | 28 |
| MS-NT | 6 | MS-SN | 21 |
| MS-NT | 7 | MS-SN | 17 |
| MS-NT | 11 | MS-SN | 19 |
| MS-NT | 9 | MS-SN | 13 |
| MS-NT | 9 | MS-SN | 16 |
| MS-NT | 8 | MS-SN | 14 |
| MS-NT | 11 | MS-SN | 19 |
|  |  | MS-SN | 21 |

The table shows the total number of c-Fos immunoreactive cells counted in the nucleus accumbens shell in both hemispheres of each rat brain coronal section. CS-NT, Control Subjects-No Test group; MS-NT, Maternal Separation-No Test group; CS-SN, Control Subjects-Social Novelty Group; MS-SN, Maternal Separation-Social Novelty group.

**Table 3.** Dataset of the number of c-Fos immunoreactive cells in the nucleus accumbens core of the different experimental groups.

| Group | Number of c-Fos positive cells | Group | Number of c-Fos positive cells |
| --- | --- | --- | --- |
| CG-NT | 10 | CG-SN | 13 |
| CG-NT | 11 | CG-SN | 15 |
| CG-NT | 9 | CG-SN | 33 |
| CG-NT | 8 | CG-SN | 25 |
| CG-NT | 8 | CG-SN | 25 |
| CG-NT | 8 | CG-SN | 23 |
| CG-NT | 9 | CG-SN | 36 |
| CG-NT | 10 | CG-SN | 25 |
| CG-NT | 10 | CG-SN | 17 |
| CG-NT | 8 | CG-SN | 23 |
| CG-NT | 6 | CG-SN | 35 |
| CG-NT | 9 | CG-SN | 24 |
| CG-NT | 9 | CG-SN | 22 |
| CG-NT | 9 | CG-SN | 20 |
| CG-NT | 6 | CG-SN | 30 |
| CG-NT | 7 | CG-SN | 51 |
| CG-NT | 4 | CG-SN | 60 |
| CG-NT | 8 | CG-SN | 75 |
| CG-NT | 14 | CG-SN | 54 |
| CG-NT | 9 | CG-SN | 46 |
| CG-NT | 9 | CG-SN | 94 |
| CG-NT | 10 | CG-SN | 78 |
| CG-NT | 12 | CG-SN | 64 |
| CG-NT | 4 | CG-SN | 77 |
| CG-NT | 4 | CG-SN | 91 |
| CG-NT | 8 | CG-SN | 84 |
| CG-NT | 7 | CG-SN | 88 |
| CG-NT | 6 | CG-SN | 87 |
| CG-NT | 10 | MS-SN | 20 |
| CG-NT | 6 | MS-SN | 12 |
| CG-NT | 9 | MS-SN | 18 |
| CG-NT | 3 | MS-SN | 12 |
| CG-NT | 2 | MS-SN | 10 |
| CG-NT | 9 | MS-SN | 12 |
| CG-NT | 6 | MS-SN | 5 |
| CG-NT | 4 | MS-SN | 9 |
| CG-NT | 10 | MS-SN | 11 |
| CG-NT | 9 | MS-SN | 13 |
| CG-NT | 11 | MS-SN | 13 |
| CG-NT | 8 | MS-SN | 5 |
| MS-NT | 7 | MS-SN | 11 |
| MS-NT | 9 | MS-SN | 11 |
| MS-NT | 7 | MS-SN | 16 |
| MS-NT | 10 | MS-SN | 17 |
| MS-NT | 10 | MS-SN | 25 |
| MS-NT | 10 | MS-SN | 36 |
| MS-NT | 8 | MS-SN | 14 |
| MS-NT | 8 | MS-SN | 13 |
| MS-NT | 8 | MS-SN | 43 |
| MS-NT | 10 | MS-SN | 21 |
| MS-NT | 11 | MS-SN | 15 |
| MS-NT | 10 | MS-SN | 12 |
| MS-NT | 10 | MS-SN | 9 |
| MS-NT | 4 | MS-SN | 21 |
| MS-NT | 15 | MS-SN | 16 |
| MS-NT | 12 | MS-SN | 25 |
| MS-NT | 8 | MS-SN | 30 |
| MS-NT | 17 | MS-SN | 22 |
| MS-NT | 9 | MS-SN | 15 |
| MS-NT | 12 | MS-SN | 19 |
| MS-NT | 7 | MS-SN | 16 |
| MS-NT | 9 | MS-SN | 15 |
| MS-NT | 5 | MS-SN | 25 |
| MS-NT | 4 | MS-SN | 28 |
| MS-NT | 9 | MS-SN | 45 |
| MS-NT | 5 | MS-SN | 63 |
| MS-NT | 4 | MS-SN | 68 |
| MS-NT | 5 | MS-SN | 43 |
| MS-NT | 2 | MS-SN | 37 |
| MS-NT | 2 |  |  |
| MS-NT | 4 |  |  |
| MS-NT | 10 |  |  |
| MS-NT | 12 |  |  |
| MS-NT | 7 |  |  |
| MS-NT | 15 |  |  |
| MS-NT | 7 |  |  |
| MS-NT | 5 |  |  |
| MS-NT | 4 |  |  |
| MS-NT | 4 |  |  |
| MS-NT | 22 |  |  |

The table shows the total number of c-Fos immunoreactive cells counted in the nucleus accumbens core in both hemispheres of each rat brain coronal section. CS-NT, Control Subjects-No Test group; MS-NT, Maternal Separation-No Test group; CS-SN, Control Subjects-Social Novelty Group; MS-SN, Maternal Separation-Social Novelty group.

**Table 4.** Dataset of the number of c-Fos immunoreactive cells in the lateral septum of the different experimental groups.

| Group | Number of c-Fos positive cells | Group | Number of c-Fos positive cells |
| --- | --- | --- | --- |
| CG-NT | 2 | CG-SN | 8 |
| CG-NT | 3 | CG-SN | 11 |
| CG-NT | 3 | CG-SN | 15 |
| CG-NT | 0 | CG-SN | 22 |
| CG-NT | 6 | CG-SN | 33 |
| CG-NT | 4 | CG-SN | 23 |
| CG-NT | 9 | CG-SN | 24 |
| CG-NT | 5 | CG-SN | 36 |
| CG-NT | 12 | CG-SN | 34 |
| CG-NT | 10 | CG-SN | 27 |
| CG-NT | 6 | CG-SN | 30 |
| CG-NT | 5 | CG-SN | 32 |
| CG-NT | 10 | CG-SN | 27 |
| CG-NT | 6 | CG-SN | 38 |
| CG-NT | 4 | CG-SN | 39 |
| CG-NT | 3 | CG-SN | 26 |
| CG-NT | 3 | CG-SN | 23 |
| CG-NT | 8 | CG-SN | 17 |
| CG-NT | 5 | CG-SN | 38 |
| CG-NT | 4 | CG-SN | 39 |
| CG-NT | 8 | CG-SN | 26 |
| CG-NT | 6 | CG-SN | 18 |
| CG-NT | 7 | CG-SN | 23 |
| CG-NT | 6 | CG-SN | 26 |
| CG-NT | 7 | CG-SN | 25 |
| CG-NT | 7 | CG-SN | 18 |
| CG-NT | 11 | CG-SN | 22 |
| CG-NT | 7 | CG-SN | 20 |
| CG-NT | 9 | CG-SN | 22 |
| CG-NT | 5 | CG-SN | 19 |
| CG-NT | 8 | CG-SN | 30 |
| CG-NT | 7 | CG-SN | 21 |
| CG-NT | 3 | CG-SN | 16 |
| CG-NT | 4 | CG-SN | 23 |
| CG-NT | 7 | MS-SN | 20 |
| CG-NT | 4 | MS-SN | 18 |
| CG-NT | 7 | MS-SN | 16 |
| CG-NT | 5 | MS-SN | 15 |
| CG-NT | 8 | MS-SN | 5 |
| MS-NT | 9 | MS-SN | 4 |
| MS-NT | 2 | MS-SN | 0 |
| MS-NT | 5 | MS-SN | 6 |
| MS-NT | 8 | MS-SN | 9 |
| MS-NT | 7 | MS-SN | 4 |
| MS-NT | 7 | MS-SN | 6 |
| MS-NT | 5 | MS-SN | 4 |
| MS-NT | 6 | MS-SN | 25 |
| MS-NT | 8 | MS-SN | 10 |
| MS-NT | 3 | MS-SN | 13 |
| MS-NT | 5 | MS-SN | 13 |
| MS-NT | 6 | MS-SN | 11 |
| MS-NT | 10 | MS-SN | 19 |
| MS-NT | 4 | MS-SN | 8 |
| MS-NT | 7 | MS-SN | 20 |
| MS-NT | 5 | MS-SN | 9 |
| MS-NT | 8 | MS-SN | 7 |
| MS-NT | 8 | MS-SN | 15 |
| MS-NT | 6 | MS-SN | 13 |
| MS-NT | 9 | MS-SN | 10 |
| MS-NT | 5 | MS-SN | 16 |
| MS-NT | 4 | MS-SN | 14 |
| MS-NT | 6 | MS-SN | 17 |
| MS-NT | 8 | MS-SN | 16 |
| MS-NT | 7 | MS-SN | 14 |
| MS-NT | 5 | MS-SN | 13 |
| MS-NT | 6 | MS-SN | 22 |
| MS-NT | 5 | MS-SN | 17 |
| MS-NT | 6 | MS-SN | 21 |
| MS-NT | 4 | MS-SN | 13 |
| MS-NT | 3 | MS-SN | 12 |
| MS-NT | 5 | MS-SN | 9 |
| MS-NT | 6 | MS-SN | 14 |
| MS-NT | 7 | MS-SN | 11 |
| MS-NT | 8 | MS-SN | 16 |
| MS-NT | 10 |  |  |

The table shows the total number of c-Fos immunoreactive cells counted in the lateral septum in both hemispheres of each rat brain coronal section. CS-NT, Control Subjects-No Test group; MS-NT, Maternal Separation-No Test group; CS-SN, Control Subjects-Social Novelty Group; MS-SN, Maternal Separation-Social Novelty group.

**Table 5.** Dataset of the number of c-Fos immunoreactive cells in the paraventricular nucleus of the hypothalamus of the different experimental groups.

| Group | Number of c-Fos positive cells | Group | Number of c-Fos positive cells |
| --- | --- | --- | --- |
| CG-NT | 5 | CG-SN | 8 |
| CG-NT | 2 | CG-SN | 8 |
| CG-NT | 3 | CG-SN | 9 |
| CG-NT | 4 | CG-SN | 6 |
| CG-NT | 2 | CG-SN | 7 |
| CG-NT | 5 | CG-SN | 22 |
| CG-NT | 3 | CG-SN | 15 |
| CG-NT | 4 | CG-SN | 16 |
| CG-NT | 1 | CG-SN | 20 |
| CG-NT | 3 | CG-SN | 18 |
| CG-NT | 7 | CG-SN | 19 |
| CG-NT | 5 | CG-SN | 19 |
| CG-NT | 5 | CG-SN | 11 |
| CG-NT | 4 | CG-SN | 14 |
| CG-NT | 2 | CG-SN | 9 |
| CG-NT | 7 | CG-SN | 7 |
| CG-NT | 5 | CG-SN | 30 |
| CG-NT | 5 | CG-SN | 17 |
| CG-NT | 4 | CG-SN | 26 |
| CG-NT | 9 | CG-SN | 12 |
| CG-NT | 2 | CG-SN | 14 |
| CG-NT | 7 | CG-SN | 17 |
| CG-NT | 5 | CG-SN | 13 |
| CG-NT | 5 | CG-SN | 20 |
| CG-NT | 10 | CG-SN | 25 |
| CG-NT | 5 | CG-SN | 19 |
| CG-NT | 7 | CG-SN | 18 |
| CG-NT | 3 | CG-SN | 27 |
| CG-NT | 4 | CG-SN | 20 |
| CG-NT | 7 | CG-SN | 11 |
| CG-NT | 8 | CG-SN | 67 |
| CG-NT | 5 | CG-SN | 29 |
| CG-NT | 5 | MS-SN | 6 |
| CG-NT | 7 | MS-SN | 10 |
| CG-NT | 6 | MS-SN | 16 |
| MS-NT | 3 | MS-SN | 5 |
| MS-NT | 4 | MS-SN | 16 |
| MS-NT | 5 | MS-SN | 17 |
| MS-NT | 2 | MS-SN | 13 |
| MS-NT | 2 | MS-SN | 18 |
| MS-NT | 5 | MS-SN | 20 |
| MS-NT | 5 | MS-SN | 16 |
| MS-NT | 5 | MS-SN | 12 |
| MS-NT | 7 | MS-SN | 4 |
| MS-NT | 6 | MS-SN | 10 |
| MS-NT | 5 | MS-SN | 11 |
| MS-NT | 3 | MS-SN | 9 |
| MS-NT | 3 | MS-SN | 11 |
| MS-NT | 5 | MS-SN | 18 |
| MS-NT | 5 | MS-SN | 20 |
| MS-NT | 7 | MS-SN | 8 |
| MS-NT | 4 | MS-SN | 31 |
| MS-NT | 8 | MS-SN | 13 |
| MS-NT | 8 | MS-SN | 8 |
| MS-NT | 3 | MS-SN | 11 |
| MS-NT | 9 | MS-SN | 9 |
| MS-NT | 3 | MS-SN | 10 |
| MS-NT | 4 | MS-SN | 11 |
| MS-NT | 6 | MS-SN | 11 |
| MS-NT | 6 | MS-SN | 20 |
| MS-NT | 7 | MS-SN | 14 |
| MS-NT | 9 | MS-SN | 14 |
| MS-NT | 5 | MS-SN | 6 |
| MS-NT | 5 | MS-SN | 4 |
| MS-NT | 7 | MS-SN | 8 |
| MS-NT | 8 | MS-SN | 9 |
| MS-NT | 6 |  |  |
| MS-NT | 6 |  |  |
| MS-NT | 5 |  |  |
| MS-NT | 2 |  |  |
| MS-NT | 5 |  |  |
| MS-NT | 4 |  |  |
| MS-NT | 4 |  |  |
| MS-NT | 2 |  |  |

The table shows the total number of c-Fos immunoreactive cells counted in the paraventricular nucleus of the hypothalamus in both hemispheres of each rat brain coronal section. CS-NT, Control Subjects-No Test group; MS-NT, Maternal Separation-No Test group; CS-SN, Control Subjects-Social Novelty Group; MS-SN, Maternal Separation-Social Novelty group.

**Table 6.** Dataset of the number of c-Fos immunoreactive cells in the medial prefrontal cortex of the different experimental groups.

| Group | Number of c-Fos positive cells | Group | Number of c-Fos positive cells |
| --- | --- | --- | --- |
| CG-NT | 5 | CG-SN | 147 |
| CG-NT | 13 | CG-SN | 143 |
| CG-NT | 5 | CG-SN | 172 |
| CG-NT | 3 | CG-SN | 232 |
| CG-NT | 2 | CG-SN | 126 |
| CG-NT | 5 | CG-SN | 250 |
| CG-NT | 8 | CG-SN | 171 |
| CG-NT | 7 | CG-SN | 227 |
| CG-NT | 3 | CG-SN | 54 |
| CG-NT | 2 | CG-SN | 49 |
| CG-NT | 8 | CG-SN | 32 |
| CG-NT | 3 | CG-SN | 35 |
| CG-NT | 13 | CG-SN | 74 |
| CG-NT | 6 | CG-SN | 60 |
| CG-NT | 9 | CG-SN | 56 |
| CG-NT | 10 | CG-SN | 14 |
| CG-NT | 9 | CG-SN | 156 |
| CG-NT | 7 | CG-SN | 132 |
| CG-NT | 9 | CG-SN | 87 |
| CG-NT | 8 | CG-SN | 177 |
| CG-NT | 11 | CG-SN | 77 |
| CG-NT | 9 | CG-SN | 110 |
| CG-NT | 6 | CG-SN | 127 |
| CG-NT | 9 | CG-SN | 94 |
| CG-NT | 12 | CG-SN | 88 |
| CG-NT | 9 | CG-SN | 79 |
| CG-NT | 7 | CG-SN | 114 |
| CG-NT | 13 | CG-SN | 67 |
| CG-NT | 9 | CG-SN | 44 |
| CG-NT | 10 | CG-SN | 58 |
| MS-NT | 4 | CG-SN | 99 |
| MS-NT | 3 | MS-SN | 37 |
| MS-NT | 8 | MS-SN | 24 |
| MS-NT | 0 | MS-SN | 40 |
| MS-NT | 14 | MS-SN | 1 |
| MS-NT | 6 | MS-SN | 3 |
| MS-NT | 3 | MS-SN | 18 |
| MS-NT | 5 | MS-SN | 16 |
| MS-NT | 3 | MS-SN | 7 |
| MS-NT | 3 | MS-SN | 5 |
| MS-NT | 8 | MS-SN | 4 |
| MS-NT | 3 | MS-SN | 10 |
| MS-NT | 5 | MS-SN | 4 |
| MS-NT | 4 | MS-SN | 3 |
| MS-NT | 3 | MS-SN | 74 |
| MS-NT | 11 | MS-SN | 71 |
| MS-NT | 7 | MS-SN | 86 |
| MS-NT | 14 | MS-SN | 116 |
| MS-NT | 8 | MS-SN | 63 |
| MS-NT | 16 | MS-SN | 125 |
| MS-NT | 8 | MS-SN | 86 |
| MS-NT | 7 | MS-SN | 112 |
| MS-NT | 7 | MS-SN | 27 |
| MS-NT | 8 | MS-SN | 47 |
| MS-NT | 10 | MS-SN | 42 |
| MS-NT | 11 | MS-SN | 16 |
|  |  | MS-SN | 74 |
|  |  | MS-SN | 60 |
|  |  | MS-SN | 56 |
|  |  | MS-SN | 14 |
|  |  | MS-SN | 40 |
|  |  | MS-SN | 17 |
|  |  | MS-SN | 20 |
|  |  | MS-SN | 90 |
|  |  | MS-SN | 71 |
|  |  | MS-SN | 55 |
|  |  | MS-SN | 113 |

The table shows the total number of c-Fos immunoreactive cells counted in the medial prefrontal cortex in both hemispheres of each rat brain coronal section. CS-NT, Control Subjects-No Test group; MS-NT, Maternal Separation-No Test group; CS-SN, Control Subjects-Social Novelty Group; MS-SN, Maternal Separation-Social Novelty group.

**Table 7.** Dataset of the total social investigation time of the experimental groups exposed to social novelty.

| **Group** | **CG-SN** | | | **MS-SN** | | |
| --- | --- | --- | --- | --- | --- | --- |
| **Subject** | **Total social investigation time (seconds)** | **Social approach frequency** | **Social approach latency** | **Total social investigation time (seconds)** | **Social approach frequency** | **Social approach latency** |
| 1 | 58.06 | 13 | 10 | 52.21 | 11 | 47 |
| 2 | 106.49 | 12 | 16 | 31.92 | 7 | 65 |
| 3 | 48.78 | 9 | 7 | 16.86 | 8 | 57 |
| 4 | 73.39 | 13 | 21 | 16.86 | 8 | 53 |
| 5 | 74.29 | 10 | 4.87 | 27.05 | 8 | 44 |
| 6 | 46.25 | 10 | 10.62 | 36.88 | 6 | 63 |
| 7 | 86.55 | 9 | 35.47 | 58.85 | 9 | 55 |
| 8 | 50.68 | 10 | 9.09 | 64.6 | 8 | 48 |
| 9 | 70.69 | 9 | 2.42 | 30.81 | 6 | 52 |
| 10 | 96.74 | 12 | 3.36 | 56.62 | 9 | 50 |

Dataset of the total social investigation time (seconds), social approach and social approach latency that subjects of the experimental groups exposed to social novelty exhibited during the social novelty tests. CS-SN, Control Subjects-Social Novelty Group; MS-SN, Maternal Separation-Social Novelty group.
